# First isolation and genotyping of *Bartonella henselae* from a cat living with a patient with cat scratch disease in Southeast Europe

**DOI:** 10.1186/s12879-019-3929-z

**Published:** 2019-04-02

**Authors:** Maja Stepanić, Sanja Duvnjak, Irena Reil, Silvio Špičić, Gordan Kompes, Relja Beck

**Affiliations:** Department for Bacteriology and Parasitology, Croatian Veterinary Institute, Savska cesta 143, 10000 Zagreb, Croatia

**Keywords:** Cat-scratch disease, *Bartonella henselae*, Child, Cat, Culture, Genotyping, MLST, Croatia

## Abstract

**Background:**

The bacterial genus *Bartonella* is distributed worldwide and poses a public health risk. Cat-scratch disease caused by *B. henselae* in Croatia was first described in 1957. It is present throughout the country: a survey of serum samples from 268 Croatian patients with lymphadenopathy showed that 37.7% had IgG antibodies. Despite this prevalence, we are unaware of reports of *Bartonella* culturing from infected humans or cats in Croatia or elsewhere in southeast Europe.

**Case presentation:**

Here we describe the diagnosis of a 12-year-old child with lymphadenopathy in Croatia with cat-scratch disease based on antibody detection and clinical signs, and the subsequent culturing and genotyping of *B.henselae* from the cat’s blood. The *B. henselae* isolate was grown on different blood agar plates and its identity was confirmed based on polymerase chain reaction **(**PCR) amplification of 16S ribosomal deoxyribonucleic acid (16S rDNA) and sequencing. Multi-locus sequence typing (MLST) identified the strain genotype as sequence type 5, commonly found zoonotic *B. henselae* strain in cats. The child recovered after azithromycin therapy, and *B. henselae* in the cat was eliminated within three months after doxycycline treatment.

**Conclusions:**

This is, to our knowledge, the first report of *B. henselae* culturing and MLST-based genotyping from cat’s blood in southeast Europe. Our ability to detect *B. henselae* in blood through culturing but not PCR suggests that the prevalence of infected cats with low bacteremia is very high, suggesting the need to develop faster, more sensitive detection assays.

## Background

The bacterial genus *Bartonella* is distributed worldwide and poses a public health risk [1]. More than 20 species cause infections in specific mammalian reservoir hosts; *Bartonella henselae,* for example, is one of the most frequent causes of zoonoses acquired from companion animals in industrialized countries. *B. henselae* is a pleomorphic, aerobic, Gram-negative bacterium that causes cat-scratch disease, which involves chronic lymphadenopathy and affects predominantly children and adolescents [2]. Domestic cats, especially young cats and kittens, are the primary reservoirs of *B. henselae;* up to 40% of domestic cats may be infected, and infections can be difficult to detect because no clinical signs may be observed even more than one year after infection [3]. Cats can infect humans directly with *B. henselae* through scratching and biting [1, 2, 4] or licking. On rare occasions, humans can be infected through bites of *Ctenocephalides felis*, a cat flea [1] that transmits *B. henselae* within cat populations [5].

Cat-scratch disease in Croatia was first described in 1957 [6], and since then only a few cases have been reported in the country. These cases were atypical because the clinical signs did not include peripheral lymphadenopathy but rather pancreatic duodenal lymphadenitis, fever, and abdominal pain [7] or osteomyelitis of the right humerus [8]. The presence of *B. henselae* in these patients was deduced from the clinical presentation, epidemiological history and presence of anti-*B. henselae* antibodies based on an indirect immunofluorescence assay (IFA). *B. henselae* is likely to be present throughout the country, since a survey of serum samples from 268 Croatian patients with lymphadenopathy showed that 37.7% patients had IgG antibodies against *B. henselae* [9]. Over a quarter of patients (28.3%) in that study had IgM antibodies, indicating acute infection. Another study in Croatia showed even higher prevalence of IgG antibodies among healthy adults (31 of 54, 57.4%) and healthy children (19 of 46, 41.3%) [6]. Despite this prevalence, we are unaware of reports of *Bartonella* sp. culturing from infected humans or cats in Croatia or elsewhere in southeast Europe.

In the absence of a consensus standard for diagnosing cat-scratch disease [10, 11], the best initial diagnostic tests are considered to be serological methods, such as indirect fluorescence or enzyme-linked immunosorbent assay [12], while the gold standard is culturing *Bartonella* sp. from the blood or tissues of infected humans or cats [13–16], followed by molecular characterization [15, 16]. However, culturing *Bartonella* sp. from humans and animals remains challenging [16–19] and has yet to be optimized [19, 20].

Here we describe the culture and genotyping of *B. henselae* from cat’s blood in Croatia, which appears to be the first such report from this country and, more broadly, from southeast Europe. The bacterium was cultured on various agar plates, and the strain was identified using multi-locus sequence typing (MLST).

## Case presentation

A12-year-old boy living in Zagreb, Croatia presented at a large pediatric clinic in the Croatian capital of Zagreb with acute enlargement of a regional lymph nodes. He was afebrile and reported pain in the axillar and antebrachial regions of the left arm that had persisted for the preceding three days. Palpation revealed a painful formation measuring approximately 1 × 1.5 cm in medial on the lower third of the left upper arm. A few smaller lymph nodes, the largest being 1 cm, were palpable in the left axillae. The patient did not show or report any other clinical signs. All haematological and biochemical parameters were within physiological ranges, including CRP. Cat-scratch disease was suspected based on anamnesis and clinical findings. Blood was taken for serological testing, and a 5-day regimen of azithromycin (500 mg/day) was prescribed. Ultrasonography of the left axillae and upper arm showed lymphadenopathy typical of cat-scratch disease. Ultrasonography performed at a private clinic revealed approximately 10 smaller lymph nodes and a few larger ones (17–27 mm) showing homogeneous cortex thickness on the left and no obvious differences from the contralateral region. IFAs were performed using a commercial kit (Focus Diagnostics, USA) in the Department of Clinical Microbiology, University Hospital for Infectious Diseases “Dr Fran Mihaljević” (Zagreb, Croatia), and the assays revealed a titer of 1:512 for IgG antibodies against *B. henselae* and 1:128 for IgG antibodies against *B. quintana* (positivity defined as ≥1:64 according to the manufacturer’s instructions). The assays were negative for IgM antibodies against both bacteria. Following azithromycin treatment, the patient showed improved status, no pain, and normal-sized lymph nodes in the left upper arm.

The patient’s parents indicated that they owned one cat and one dog, and that the patient played frequently with the cat. The cat was an apparently healthy, 10-month-old female British shorthair that lived primarily indoors but had free access to a yard; the cat had a history of flea infestation. When a diagnosis of cat-scratch disease was suspected, a sample of the cat’s blood was collected at a private veterinary clinic and delivered to the Croatian Veterinary Institute for analysis. Upon isolation of *B. henselae* from the blood (see below), the cat was treated for 3 weeks with doxycycline (10 mg/kg body weight p/o every 12 h) [21, 22], and the patient’s parents were advised to protect the cat against ectoparasites.

Both when a diagnosis of cat-scratch disease was suspected and again three months later (following doxycycline treatment), cat’s blood was collected in K2-EDTA tubes (Vacuette, Greiner) and stored overnight at − 18 °C to lyse erythrocytes and release bacteria. The frozen blood was thawed at room temperature, and aliquots (200 μL) were plated in duplicate onto Tryptic soy agar with 5% defibrinated sheep blood (TSA) and Brain heart agar with 5% defibrinated rabbit blood (BH). Both agar bases were purchased from Merck (Darmstadt, Germany) and used to prepare “ready-to-use” plates. Blood was inoculated onto the plates by simultaneously tilting them at a 45-degree angle and rotating them, allowing the blood to flow across the agar without the need for an inoculating loop or mechanical streaking [23, 24]. The plates were then incubated, agar side down, at 37 °C in a humidified atmosphere of 8% CO_2_ [19, 25]. At three days after inoculation, plates were inverted to sit agar side up. At six days after inoculation, single colonies from each type of plate were picked for molecular analysis (see next section). At the same time, single colonies from two TSA plates were subcultured onto two fresh TSA plates, while single colonies from one BH plate were subcultured onto a single fresh BH plate. Cat’s blood after doxycycline treatment was plated onto two fresh TSA and BH plates. Subcultured and plates inoculated after doxycycline treatment were incubated under the same conditions as the first set of plates. Colonies from plates that were subsequently identified as *Bartonella* spp. by PCR were stored at − 80 °C in *Brucella* broth supplemented with 10% glycerol (*v*/v) [26].

DNA was extracted from 200 μl EDTA blood and from six cultures using the QIAcube automated DNA isolation system and QiaAmp DNAmini QIAcube Kit, according to the manufacturer’s instructions for blood and tissue (Qiagen, Hilden, Germany). In order to determine whether isolates belonged to the genus *Bartonella,* DNA from six culture isolates were used as template in conventional PCR targeting 16S rRNA (16S-R as forward primer: F 5′-GCC YCC TTG CGG TTA GCA CAG CA-3′ and P24Emod as reverse primer: R 5′-CCT TCA GTT MGG CTG GAT C-3′) as well as the intergenic transcribed spacer between the 16S and 23S rRNA genes (Bart/16-23F as forward primer: 5′-TTG ATA AGC GTG AGG TCG GAG G-3′ and Bart/16-23R as reverse primer: 5′-CAA AGC AGG TGC TCT CCC AG-3′) [27].

The DNA extracted from cultures served as template for MLST at the following nine loci in housekeeping genes [28]: 16S rRNA gene, *batR, eno, ftsZ, gltA, groEL, nlpD, ribC,* and *rpoB*. MLST sequences were assembled, processed and compared using BioNumerics 7.6 software (Applied Maths, Belgium), which identified alleles and sequence types based on published profiles [28]. Alleles were assigned 9-digit numerical codes and compared using the categorical coefficient and hierarchical clustering using the unweighted pair group method with arithmetic mean (UPGMA) method. MLST results were compared with sequence types (STs) in the *B. henselae* database (http://bhenselae.mlst.net/) [29].

At three days after inoculation, plates did not show visible colonies, although a few plates contained “shiny islets” suggestive of initial bacterial growth. At six days after inoculation, two TSA plates and one BH plate showed visible *Bartonella-*like colonies (Table 1). Upon subculturing onto two fresh TSA plates and one fresh BH plate, growth was visible on all three plates after four days, and it was abundant after six days (Table 1). A blood sample taken from the cat after doxycycline treatment did not give rise to bacterial growth even after one month of incubation.

**Table 1:** Time course of growth of *Bartonella* spp. and PCR confirmation after blood of the infected cat was inoculated onto agar plates (primary culture) or subcultured (secondary culture)

Plate no.	Agar	Culture	Result after days of incubation			
			3	4	6	7
1	TSA	Primary	Initial growth (SI)		Visible growth	PCR+
		Secondary	–	Initial growth	Visible growth	PCR+
2	TSA	Primary	Initial growth (SI)		Visible growth	PCR+
		Secondary	–	Initial growth	Visible growth	PCR+
3	BH	Primary	Initial growth (SI)		Visible growth	PCR+
		Secondary	–	Initial growth	Visible growth	PCR+
4	BH	Primary	Contaminated, discarded	–	–	–
		–	–	–	–	–

**Fig. 1:**
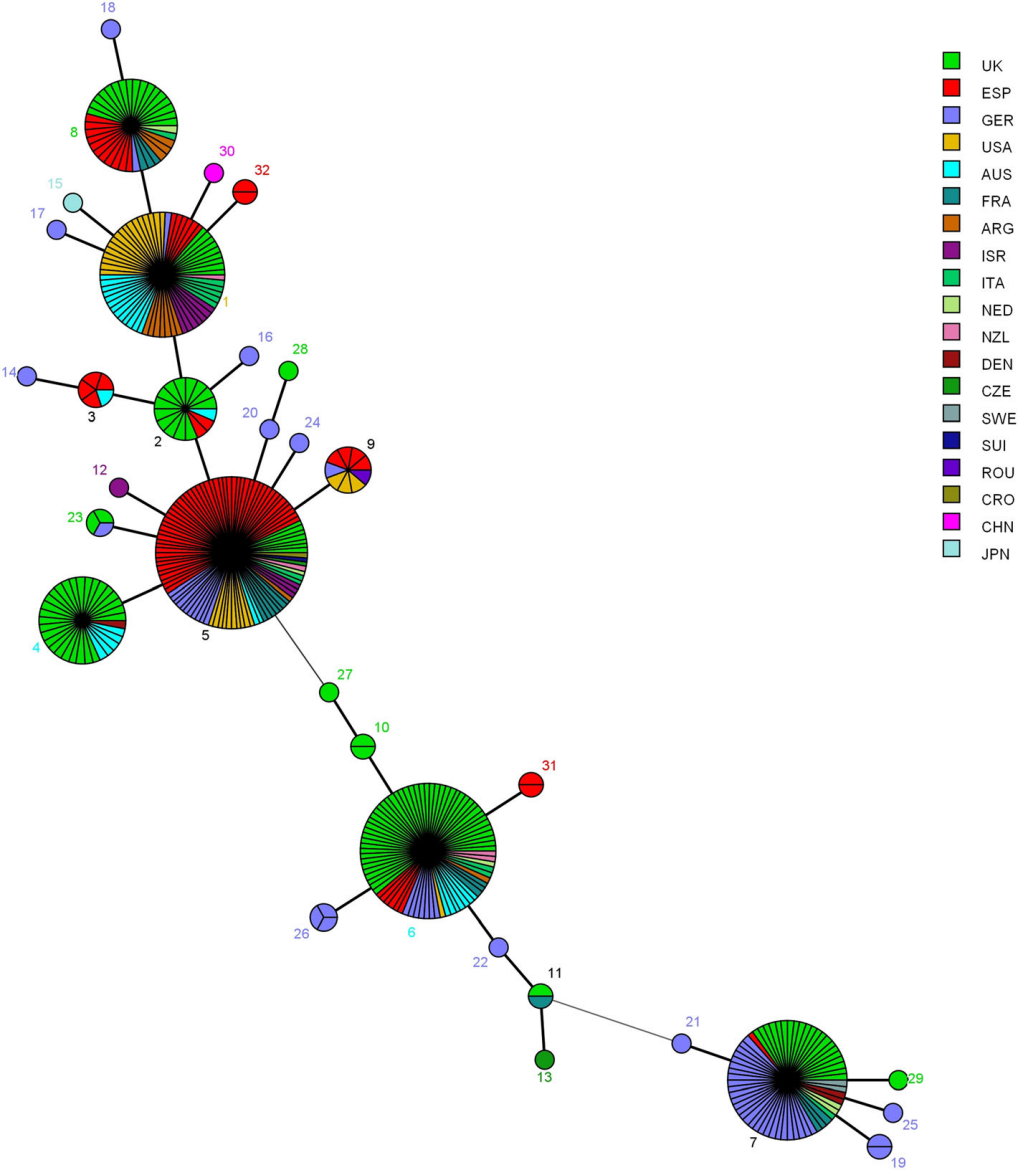
Multilocus sequence typing of *Bartonella henselae* strains present in http://bhenselae.mlst.net/ and reported in the present study (CRO). Strains are grouped according to sequence type and labeled by country of origin

PCR to amplify *Bartonella* spp. 16S rRNA and intergenic spacer regions was negative for DNA extracted from cat blood sampled before and after doxycycline treatment. Both types of PCR gave positive results when DNA was extracted from TSA and BH colonies cultured from the blood. Sequencing and BLAST analysis indicated *B.henselae.* The strain was genotyped by MLST and found to have the code 2–1–1-1-1-2-1-1-1, corresponding to genotype ST5 (Fig. 1).

## Discussion and conclusions

Cat-scratch disease typically presents as regional lymphadenopathy in people with a history of cat contact, usually kittens [30]. The disease occurs more often in younger people with flea-infested cats younger than one year, and in people who have been scratched or bitten by a cat [4]. Cat-scratch disease causes regional, mainly unilateral, lymphadenopathy that localizes to the upper extremities on the injury side in immunocompetent patients [12], with 5–9% of affected individuals developing atypical manifestations [4]. The patient in the present study fits this profile well. He had an anti-*B. henselae* titer of 1:512, while titers higher than 1:256 strongly suggest active or recent infection [12]. Noninfectious causes of lymph node enlargement, such as malignancy, were excluded [12], and azithromycin treatment proved effective for rapid resolution of lymphadenopathy [12, 30]. Since the patient met basic criteria for the diagnoses of cat-scratch disease (lymphadenopathy, seropositivity, contact with cat and exclusion of malignancy), and since the cat was confirmed to be infected with *Bartonella* spp., no additional diagnostic procedures were performed. Molecular analysis of bacterial isolates and MLST-based genotyping indicated that the cat was infected with *B. henselae*. To our knowledge, this is the first report of *B. henselae* culture from the blood of an infected cat in southeast Europe.

Our results confirm that although the genus *Bartonella* is fastidious and slow-growing, it can be cultured [31, 32], even relatively rapidly: colonies became visible after 6 days in primary culture and after 4 days in subculture. In contrast, previous work reported that primary *Bartonella* colonies became visible as soon as 3–5 days after plating but usually after 12–14 days, sometimes 45 days [23, 33] or 56 days [31], while subcultured colonies appeared after 4–10 days [16], sometimes 15 days [33]. Other studies of *Bartonella*-like colonies from inoculated cat’s blood have also reported slower first appearance than in our study, including 10–15 days on Columbia agar plates with 5% sheep blood [26], 1–2 weeks on Heart infusion agar with 10% rabbit blood [34], and 2 weeks (primary culture) or 5 days (subculture) on chocolate agar plates with 5% defibrinated sheep blood [35]. In our study, primary *B. henselae* colonies were rough and adherent, and they pitted the agar. Upon subculture, they became larger, smooth and less adherent, as described previously [33, 36].

Our results suggest that direct blood culture is more sensitive than conventional PCR of whole blood for detecting *B. henselae,* similar to the conclusions of other authors [37]. Direct blood culture also appears to be preferable to IFA-based antibody detection because most flea-exposed cats develop anti-*Bartonella* antibodies, even when they are not bacteremic [38]. On the other hand, some studies have suggested that PCR, especially nested or real-time rather than conventional PCR, can be more sensitive than culturing [16, 34]. Nevertheless, isolation by culture is encouraged for MLST genotyping because it enables broader molecular characterization and accurate species determination, and it proves that the isolate is living [16]. In the end, culture and PCR are complementary methods and so both should be used whenever possible [15, 16]. Cats that serve as chronic subclinical reservoirs with cyclic bacteremia often give false negative results by PCR [22], probably due to the small number of bacteria in the sample [37], and this may explain our negative results in the present case. MLST indicated that our *B. henselae* strain belongs to the most widespread zoonotic sequence type, ST5. Together, ST2, ST5 and ST8 account for 85% of zoonosis-associated *B. henselae* strains, according to a sampling study in the UK [39]. ST5 accounted for 20.9% of 182 *B. henselae* isolates from 12 countries on three continents, and it has been reported in humans and cats in Europe, the US and Australia [40]. MLST of 78 feline and 53 human isolates in Spain found that types ST5, ST6 and ST9 were associated with felines, while humans were most often infected with ST1, ST5 or ST8. ST5 in that study accounted for 53.7% of *B. henselae* infections in humans and 61.5% of infections in cats [41], suggesting that it is the predominant variant infecting felines and human beings. Our results support previous knowledge about global prevalence of zoonotic genotype *B. henselae* ST5.

The most frequent route of *B. henselae* transmission between cats is via the flea *Ctenocephalides felis* [4, 31] or their feces [13], in which bacteria can survive for at least nine days [14]. Flea control appears to be the only effective way to prevent infection of cats. Using antibiotics to treat clinically healthy cats infected with *B. henselae* is controversial, since bacteremia can persist for 22–33 weeks in experimentally infected cats [42–44]. Nevertheless, the cat in the present study was treated with doxycycline because she was bacteremic and because the owners requested it.

The present results show, for the first time, that zoonotic *B.henselae* strain ST5 is present in southeast Europe. Further studies are needed to investigate *B. henselae* prevalence in humans and cats, and correlation between isolates based on culturing and MLST. Our ability to detect *B. henselae* in blood through culturing but not PCR highlights the likely existence of infected cats with low bacteremia, suggesting the need to develop faster, more sensitive detection assays.

## Abbreviations

16S rDNA: 16S ribosomal deoxyribonucleic acid
*batR*: Two-component regulator
BH: Brain heart agar
CRP: C-reactive protein
*eno*: enolase
*ftsZ*: Cell division protein
*gltA*: Citrate synthase
*groEL*: Hsp60 chaperone
IFA: Immunofluorescence assay
K2-EDTA: dipotassium ethylenediaminetetraacetic acid
MLST: multi-locus sequence typing
*nlpD*: Cell surface glycoprotein
PCR: polymerase chain reaction
*ribC*: Riboflavin synthase
*rpoB*: RNA polymerase beta subunit
SI: “shiny islet” incipient colonies
ST: Sequence type
TSA: Tryptic soy agar
UPGMA: Unweighted pair group method with arithmetic mean
